# Non-Simian Foamy Viruses: Molecular Virology, Tropism and Prevalence and Zoonotic/Interspecies Transmission

**DOI:** 10.3390/v5092169

**Published:** 2013-09-13

**Authors:** Timo Kehl, Juan Tan, Magdalena Materniak

**Affiliations:** 1 German Cancer Research Center, INF242, Heidelberg 69120, Germany; 2 College of Life Sciences, Nankai University, 94 Weijin Road, Tianjin 300071, China; E-Mail: juantan@nankai.edu.cn; 3 Department of Biochemistry, National Veterinary Research Institute, Partyzantow Ave. 57, Pulawy 24-100, Poland; E-Mail: magdalena.materniak@piwet.pulawy.pl

**Keywords:** foamy virus, interspecies transmission, FV tropism, zoonotic potential, animal FVs

## Abstract

Within the field of retrovirus, our knowledge of foamy viruses (FV) is still limited. Their unique replication strategy and mechanism of viral persistency needs further research to gain understanding of the virus-host interactions, especially in the light of the recent findings suggesting their ancient origin and long co-evolution with their nonhuman hosts. Unquestionably, the most studied member is the primate/prototype foamy virus (PFV) which was originally isolated from a human (designated as human foamy virus, HFV), but later identified as chimpanzee origin; phylogenetic analysis clearly places it among other Old World primates. Additionally, the study of non-simian animal FVs can contribute to a deeper understanding of FV-host interactions and development of other animal models. The review aims at highlighting areas of special interest regarding the structure, biology, virus-host interactions and interspecies transmission potential of primate as well as non-primate foamy viruses for gaining new insights into FV biology.

## 1. Defining Statement

We will not provide a full review of non-simian foamy virus (FV) biology (hereafter referred to as animal FVs) and their features since many of these aspects have been recently described, especially from the perspective of the prototype foamy virus (PFV) [1,2,3,4]. In this review we will describe and discuss features where studies on the animal FVs significantly contributed to our overall understanding of FV biology. Studies on animal FVs offer, due to the possibility to conduct comparably easy animal experimentation, a unique chance to analyze in details and depth the virus–host interaction at a cellular, organismal and cladal (epidemiological) level. In this context, the potential of zoonotic transmissions as well as interspecies transmissions will also be discussed. 

## 2. Classification of a Complex Group of Viruses

### 2.1. Natural History and Genome Organization

Foamy viruses, also known as *syncytial* or *spumaviruses*, represent the subfamily *Spumaretrovirinae* within the *Retroviridae* [5]. Simian FVs (SFVs) were first described by Enders and Peebles in 1954, subsequently followed by isolation from a nasopharyngeal carcinoma of an African patient, nowadays known as the end-product of a zoonotic transmission of a chimpanzee FV to a human being and originally designated human foamy virus (HFV) and currently as primate/prototypic FV (PFV) [6,7,8,9]. 

**Figure 1:**
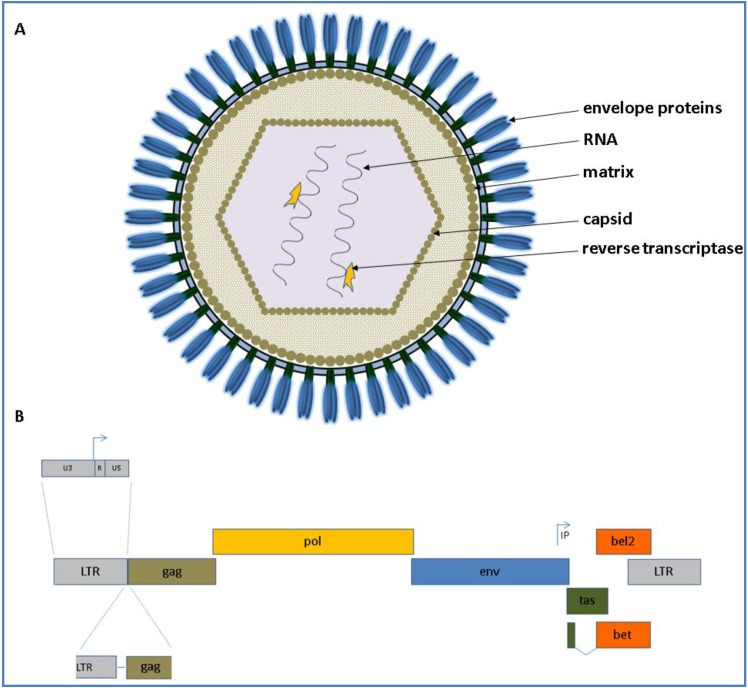
(**A**) Schematic presentation of a foamy virus (FV) virion is shown at the top. The appearance of the virion is based on current knowledge and observations of Wilk and co‑workers who analyzed PFV and feline FV (FFV) capsids by cryo electron microscopy (cEM), paired with surface plasmogen resonance (SPR) analyses [17]; (**B**) The genomic organization of an animal FV at the DNA level is shown below (from 5' to 3'). The scheme is drawn proportionally to the original length of each gene. The canonical *gag*, *pol* and *env* genes are shown in brown, yellow and blue, respectively. IP displays the internal promoter site 3' in the *env* gene. Arrows indicate transcription start sites. The overlapping open reading frames (ORFs) for *tas* and *bel2* are displayed in green and orange, respectively. The *bet* transcript is shown as a spliced product of the N-terminal *tas* and the complete *bel2* ORF.

As FVs are complex retroviruses like the lentiviral human immunodeficiency virus (HIV) and encode the canonical *gag*, *pol* and *env* genes flanked by the long terminal repeats (LTRs) and additional accessory genes designated as *tas* (former designated as *bel1*) and *bel2* open reading frames (ORF). The accessory Bet is transcribed by a spliced product of the N-terminal *tas* and the complete *bel2* ORF. The schematic appearance of a FV virion as well as its genome is depicted for all animal FVs displayed in Figure 1. 

Within the *Spumaretroviridae*, there are different types of FVs circulating within a species which may be a result of FVs’ co-evolution with their hosts, dated back for at least 60 million years [4,10,11]. Most recently, this putative coevolution was extended to 105 million years ago (mya) by Katzourakis *et al.* as this group has been able to demonstrate endogenous elements of a FV in the genome of a sloth [12]. However, FVs might have their origin even as far back as 400 mya, since Han *et al.* found an endogenous foamy-like element in the Coelacanth genome, an ancient living fossil from the Devonian period of the Paleozoic era [13]. Moreover, recent results indicate FVs in the primate *Daubentonia madagascariensis* (aye-aye) and a Chinese bat [14,15]. Regarding the recent findings on novel endogenous and exogenous FVs, one may presume that the list of FV hosts is not complete yet and there may be further unknown FVs infecting even non-mammalian vertebrate phyla. Thus, the suggestion that the host range of FVs simulate that of lentiviruses may be an oversimplification of the evolution and distribution of both retrovirus groups [1,16].

Except the FV clades and isolates shown in Table 1 which are based upon biological and molecular evidence (sequences and/or virus isolation), there have been single reports on FVs in sea lions, domestic sheep, wild ungulates and even hamsters [18,19,20,21]. Since either an isolation of the virus or a follow up of these reports are missing, future studies are required to confirm these cases. SFVs, however have already been isolated decades ago and analyzed on molecular basis (see Table 1). 

**Table 1:** Foamy virus clades/isolates and their hosts.

Clade/isolate	Host	References
SFV (incl. PFV)	simians, apes and humans	[8,10,22,23,24,25,26]
FFV	cats (domestic and wild)	[27,28,29]
BFV	cattle	[30,31,32]
EFV	horse	[33]
RaFV-1	bat	[14]
CoeEFV	coelacanth (endogeneous)	[13]
SloEFV	sloth (endogenous)	[12]
PSFVaye	*Daubentonia madagascariensis* (aye-aye)	[15]

### 2.2. Overall Genome-Wide Comparison of Animal FVs

**Figure 2:**
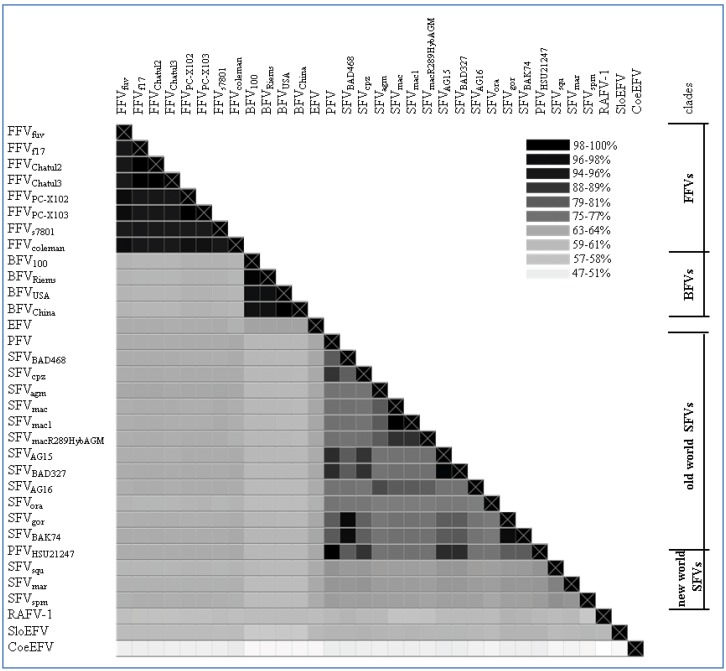
Genomic *pol* alignment of FV types and isolates with a complete *pol* sequence, excluding the bat FV RaFV-1: The alignment is visualized as a heatmap displaying the sequence identity (% of identical residues) among the different FV types/isolates as grey-scales. The color scheme is computed as a continuum of grey scale colors from light grey to black as indicated in the legend and correlates to the percentage of sequence identity as mentioned above. On the right of the heatmap a separation between the different FV clades is displayed as bars; single isolates have been left blank. Nucleotide sequence alignment was done using a progressive pairwise alignment method built in the Geneious 6.05 software tool available from Biomatters [44]. Accession numbers used for phylogeny are depicted in Table 2. Non listed FV accession numbers in Table 2, but used for the heatmap are as follows: Feline FV strains from: felis catus (*Felis silvestris catus*; Chatul2, AJ564745; Chatul3, AJ564746; and puma (*Puma concolor*; strain FFVpc-X103, KC292055); Simian FV strains from: macaque (SFVmac R289HybAGM, JN801175; SFVmac1, NC_010819); chimpanzee (*Pan troglodytes troglodytes*; strains AG15, JQ867462; BAD327, JQ867463); gorilla (*Gorilla gorilla*, strains BAK74, JQ867464; SFV BAD468, JQ867465); and monkey (*Cercopithecus nictitans*; strain AG16, JQ867466).

**Figure 3:**
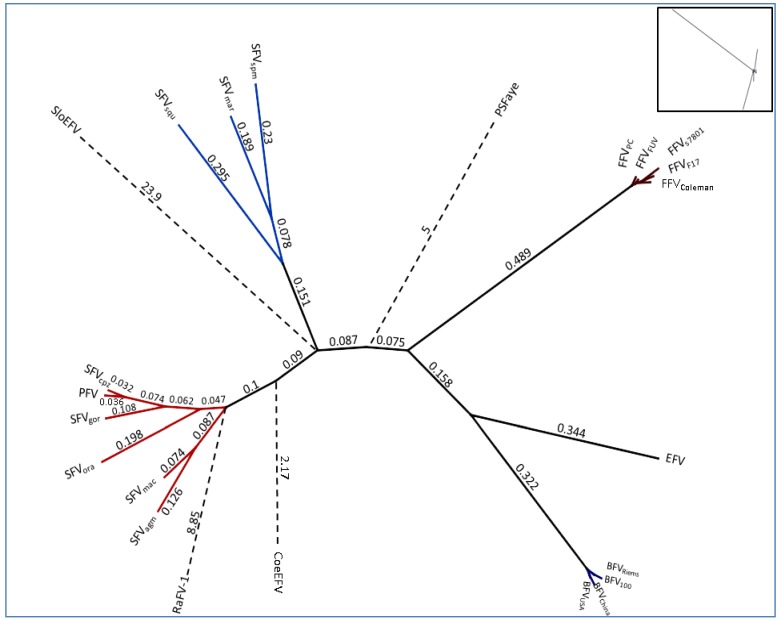
Unrooted phylogenetic tree of the complete Pol protein sequence from FV genomes listed in Table 2: For RaFV-1 and PSFVaye the available amino acid sequences were shorter, 475 and 733 aas respectively. Amino acid alignment for subsequent tree analysis was done using a global alignment with a Blosum62 matrix [44]. The phylogenetic tree was computed, using PHYML combined with the LG matrix and bootstrapping (1,000 replicates). The tree design was transformed proportional in order to display the high substitutions per site of RaFV-1, SloEFV, PSFVaye and CoeEFV. The numbers atop the branches display the substitutions per site. The insert in the right upper corner, displays the initial computed tree as an original overview for the branch lengths which are transformed for visibility’s sake for the endogenous CoeEFV, SloEFV, pSFaye and RaFV‑1 (dashed lines). Viral designations are given in Table 1. Accession numbers for most FVs are given in Table 2. The missing accession numbers are not available and sequences used can be found in the references for endogenous FVs: RaFV-1 (JQ814855.1), PSFVaye [15], CoeEFV [13], SloEFV [12].

Based on the highly conserved *pol* genomic sequence, an alignment was computed and visualized as a heatmap (Figure 2) showing the sequence identity between the different FV types and isolates listed in Table 2 and the legend of Figure 2, respectively. Furthermore, on the protein level a phylogenetic tree was computed using Pol and is shown in Figure 3 to demonstrate the overall phylogenetic relationship between the exogenous and endogenous FVs. Due to the high substitution rates and the little genetic information available for some of the FVs (aye-aye and bat FV) their branches were transposed for visibility’s sake. Additionally, further trees have been computed, backing up our findings on *pol* by using Gag, Bet, Env, Tas and Bet as basis for analysis (data not shown). 

### 2.3. Feline Foamy Virus Clade

As shown in Figure 2, the FFV clade clusters on genetic level with a sequence identity of about 94%–99%. The phylogenetic FFV distribution and relatedness of different isolates within the FFV clade was already described by Phung and co-workers in 2001 [34]. Here, full-length genomes of two FFV_PCs_ isolated from free-living North American pumas (*Puma concolor*) are included in the phylogenetic analysis [29]. Therefore, we can show for the first time a FV clade clustering similar to SFV with distant and highly related hosts and their corresponding FVs. Compared to the SFV alignment and phylogenetic tree, the FFV_PCs_ are by far closer related to the other known FFV isolates than the different SFV types are as indicated by the mean of the % identities of the sequence alignment of *pol* from FFVs (95.575%, CI from 94.83–96.32) *vs.* Old World SFVs (79.65%, CI from 78.23–81.08). 

Among the FFVs two different serotypes have been characterized: the FUV7 like FVs and the F17/951-like serotypes [35,36,37,38]. Originally, the two distinct serotypes of FFV have been identified due to differential neutralization patterns, mainly based on sequence differences in Env Su (Env surface domain) [39]. How the two serotypes evolved is currently unclear. One option would be the genetic uptake of an *env* gene or part of it of a so far unknown FFV type. This potential recombination event could be facilitated by exchange of a highly divergent env region between FUV and F17/951-like FFVs, as shown in Winkler *et al.* and personal communication with Martin Löchelt [38,40]. The recently identified FFV_PC_ exhibits the same branching node as FFV_FUV_, and therefore belongs to the FUV7 like FFVs. Furthermore, the F17/951-like serotypes form a cluster including the FFV_Coleman_, FFV_S7801_ and FFV_F17_ isolates.

Currently there are only some domestic cat FFVs and two highly related *Puma concolor* FFV isolates for phylogenetic analysis available. To combine our genetic and phylogenetic analyses with the existing theories of the evolutionary origins of the *Felidae* and the biogeographic distributions of cats, the available data pool is insufficient. To elucidate the distribution of FFVs and their virus-host interactions in more detail we need to find additional FFV species in less investigated feline hosts [41,42,43]. Nevertheless, the last common ancestor of the modern *Felidae* lived about 10.8 mya in South-East Asia, and gave rise to 37 different species so far [41,42,43]. 

**Table 2:** Accession numbers and references used for FV phylogeny.

Acronym	Description	Virus clade/isolates	Accession number
BFV**_100_**	Bovine Foamy Virus BFV100 isolate, Poland	Bovine foamy virus	JX307861.1
BFV**_China_**	Bovine Foamy Virus Geng isolate, China	Bovine foamy virus	AY134750
BFV**_Riems_**	Bovine Foamy Virus Riems isolate, Germany	Bovine foamy virus	JX307862.1
BFV**_USA_**	Bovine Foamy Virus Casey isolate, USA	Bovine foamy virus	U94514
EFV	Equine Foamy Virus isolate	Equine Foamy Virus	AF201902
FFV**_FUV_**	Feline Foamy Virus FUV ioslate, Germany	Feline foamy Virus	Y08851
FFV**_PC_**	Feline Foamy Virus Puma C. isolate, USA	Feline foamy Virus	KC292054
FFV**_F17_**	Feline Foamy Virus F17 isolate, USA	Feline foamy Virus	NC_001871.1
FFV**_Coleman_**	Feline Foamy Virus Coleman isolate, USA	Feline foamy Virus	AB052797
FFV**_S7801_**	Feline Foamy Virus S7801 isolate, Japan	Feline foamy Virus	AB052796
SFV**_agm_**	Simian foamy virus 3, Vervet monkey	Simian foamy Virus	NC_010820
SFV**_cpz_**	Simian Foamy Virus, Chimpanzee	Simian foamy Virus	U04327
SFV**_gor_**	Simian Foamy Virus, Gorilla	Simian foamy Virus	HM245790
SFV**_mac_**	Simian Foamy Virus 1, Macaque	Simian foamy Virus	X54482
SFV**_ora_**	Simian Foamy Virus, Orang Utan	Simian foamy Virus	AJ544579
PFV	Human Foamy Virus isolate	Simian foamy Virus	U21247
SFV**_spm_**	Simian Foamy Virus, Spider monkey	Simian foamy Virus	EU010385.1
SFV**_mar_**	Simian Foamy Virus, Marmoset monkey	Simian foamy Virus	GU356395
SFV**_squ_**	Simian Foamy Virus, Squirrel monkey	Simian foamy Virus	GU356394
SFV**_gor_**	Simian Foamy Virus, Gorilla	Simian foamy Virus	HM245790
SloEFV	Endogenous Foamy Virus, Sloth	Sloth Foamy Virus	[12]
CoeEFV	Endogenous Foamy Virus, Coelacanth	Coelacanth Foamy Virus	[13]
RaFV-1	Exo- or endogenous Foamy Virus, Bat	Bat Foamy Virus	[14]

Except for the FFV_PC_ isolates which have been isolated recently from *Puma concolor*, all other FFV types in Table 2 have been isolated from different types of domestic cat species. Pumas and domestic cats are closely related as the domestic cat species descended from the same branching node within the phylogenetic tree of the *Felidae* from which the pumas separated as well [41,42]. 

The high sequence identity could thus be explained by the close relationship of the hosts and the same evolutionary pressure on the FFVs during this relatively low evolutionary time frame of 3.4–6.7 mya. Another possible more likely explanation would be a more recent interspecies transmission of FFV from domestic cats to wild pumas or vice versa.

### 2.4. Ungulate Foamy Virus Clade

All analyzed BFV isolates have been taken from domestic cattle in USA, China, Poland and Germany. Since the domestic cattle has its source from the aurochs about 7,000 years ago in Europe, only minor differences in the BFV *pol* sequence are to be expected as shown in Figure 2 and Figure 3. The sequence identity fluctuates between 96%–99%. BFV isolates from China and USA (BFV_USA_ and BFV_China_) arise from the same branching node, forming a clade just as BFV_100_ (Poland) and BFV_Riems_ (Germany) form the European clade (see Table 2) [31]. If BFV and the equine foamy virus (EFV) derived from a common ancestor far back in the early Eocene epoch (~50 mya) when pre-cattle and horses first hit the ground this would be the earliest time point for a possible infection with FVs. Therefore, the low sequence identity of 65% and the phylogenetic placement of EFV isolate close to the BFV clade can be explained by, and would favor the coevolution theory of FVs with their hosts, rather than an occasional interspecies transmission.

### 2.5. Simian Foamy Virus Clade

A more extended and diffuse identity cluster is presented by the Old World monkey foamy virus types/isolates and the New World monkey foamy virus types in the current literature summarized as SFVs. Among these sequence similarities range from 64%–99% which makes SFVs the most diverse FV cluster among all known FVs. Surprisingly, simian FVs do not form a densely organized cluster as would be anticipated by their name. Phylogenetic analyses as well as alignment analyses (heatmap) show a distribution of the SFVs into two subgroups: the New World monkey FVs (SFV_sqm_, SFV_spm_ and SFV_mar_; sequence identity between 68%–72%) and the Old World monkey FVs (SFV_agm_, SFV_mac_, SFV_gor_, SFV_ora_, SFV_cpz_ and PFV; sequence identity between 73%–99%). Katzourakis and colleagues showed a phylogenetic separation of SFV_agm_, SFV_mac_, SFV_ora_ and SFV_cpz_ *vs.* SFV_spm_ [12]. Unfortunately in their investigations the clade differences did not present themselves very obvious as they were aiming to introduce the recently identified endogenous elements for FVs and additionally were using a small subset of SFVs for their analyses. A more comprehensive profile of all known New and Old World SFVs including PFV as shown in Figure 2 and Figure 3 illustrates the two entirely separated clades, closely correlating with Michael Heads evolution theory of the distribution of monkeys, mentioned below [45]. So far zoonotic transmission events of New World SFVs to humans have not been reported, thus leaving it open whether the New World SFVs have a comparable zoonotic potential like the Old World SFVs as it is proposed for SFV_gor_, SFV_cpz_ and SFV_agm_ in many publications [9,46,47,48,49]. Thus it would be interesting to study whether the lack of known New World SFV zoonoses is simply due to the lack of studies or whether it is related to the higher genetic divergence between humans and New World monkeys. Additionally, in line with the finding that a SFV_cpz_ is the origin of PFV, the latter clusters as one of the “youngest” FVs right next to the SFV_cpz_ (Figure 3) [5]. Due to its zoonotic origin and encouraging attempts for vector development, research was and still is focused on PFV. 

We believe that the status “PFV” constrains at least in part the general conceptual reasoning of FV research which means that research done on PFV is neither generally transposable to SFV nor to the even more distant animal FVs. Using the phylogenetic analyses for the SFV types, PFV does not seem to be prototypic, questioning why so much effort is put in this very special FV type. In the light of the latest reports which postulate that FVs coevolved with their hosts it seems even possible that the “real prototype FV” should be rather sought for far back in the evolutionary process.

### 2.6. “Exotic” Foamy Viruses

According to the heatmap and the unrooted phylogenetic Pol analysis the endogenous FVs and RaFV-1 show the most divergent distribution with the highest substitution rates per site as compared to FFV, BFV and SFV. The mean sequence identity of *pol* to the other FVs is 59.7% (CI 59.3–60.1) for RaFV-1, followed by SloEFV 60.27% (CI 59.77–60.77) and CoeEFV 48.51% (CI 48.13–48.9). The distant tree distribution of the endogenous FV in aye-aye, a prosimian lemur, living on Madagascar, fits into a modern evolution theory, according to which the monkeys split up not 60 but nearly 100 mya in compliance with the tectonically plate shift [45]. The separation of the tectonic plates and the following absence of genetic mixing led to the separated evolution of dry and wet nose apes the latter ones diverging into the lemurs and loris (both are Prosimians). Nevertheless, one should keep in mind that endogenous elements are rarely protected from mutations and recombination, therefore a robust delimitation of the vertices and terminal node positions within the phylogenetic tree remains to be elusive. Moreover, the theory of FV coevolution with its natural hosts might have been disrupted occasionally. A candidate for interspecies transmission would be the newly discovered RaFV-1. Bats are commonly known to be a cornucopia of pathogens which in some cases are associated with the host from early on, in other cases have been affiliated more recently by interspecies transmission. Considering the phylogeny of species and the putative coevolution of FVs with their hosts, bats could have acquired a FV as far as 50–100 mya, possibly within the same time frame when monkeys aquired SFVs. Additionally, the phylogenetic positioning of RaFV-1 within the New World SFVs, rather than within Old World SFVs, as would be expected according to their geographical distribution is questionable and might be explained by the low biostatistics likelihood for the branching node of RaFV-1. In summary, in order to come to a profound conclusion further analyses on RaFV-1 have to be performed.

### 2.7. Conservation of FV Specific Traits

The different FV types and isolates show a sequence identity mean (*pol*) of 66.66% (SD: 11.34) (see Figure 2). This overall identity reflects only faintly the highly conserved sequence pattern of the FFV and BFV clade contrasting the diverse Old World monkey SFV clade. Strikingly, the age difference of the different FVs is rather high, ranging from approximately 10,000 years (cattle, BFV) to estimated 400 mya (Coelacanth, CoeEFV). In addition to the phylogenetic relationship the conservation of typical FV-specific motifs like the internal promoter site (IP) over the whole time period of FV evolution is of special interest.

**Figure 4:**
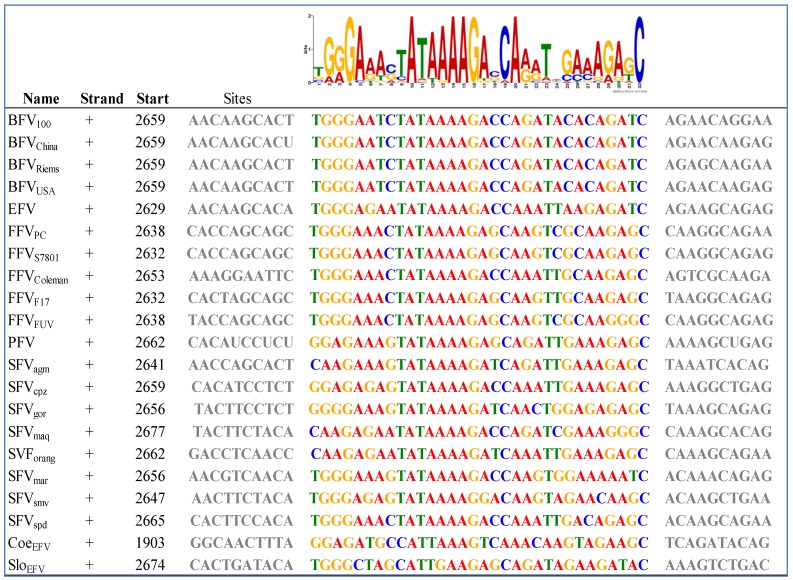
Bioinformatics-based detection of the IP TATA box and flanking sequences in exogenous and ancient endogenous FVs using the MEME (Multiple EM for Motif Elicidation) software [53]: The results obtained by MEME are displayed in a consensus sequence logo Figure on top of the individual sequences of the analyzed FVs (see Table 2). The logo presents the nucleotides by position-specific probability matrices specifying the probability of each possible letter appearing at each possible position within the motif. The nucleotides are stacked and the total height of the stack is the “information content” of that position in the motif. The height of the individual letters in a stack is the probability of the letter at that position multiplied by the total information content of the stack. Software parameters where chosen as followed: distribution of motif occurrences (zero or one per sequence), number of different motifs (20), minimum motif width (6) and maximum motif width (32).

To our knowledge there are some bioinformatics analyses on the IP of FVs but most of them are restricted to SFV and no complete IP analysis has been performed so far on all known FV species [50]. As already mentioned, the internal promoter site is located within the 3' end of *env*, upstream of the regulatory *tas* and the accessory *bel2* gene, and it is absolutely indispensable for overall FV gene expression and infectivity [50,51,52]. Using a special motif search program called MEME, we aimed at finding IP-associated *cis*-acting motifs of the IP region in all known FV species, including the ancient endogenous CoeEFV and SloEFV genomes [53]. This analysis shows that the unique FV feature of an IP in *env* must have been introduced or was already present in FVs at least 400 mya (Figure 4). 

Moreover, this motif had been conserved throughout the whole viral-host evolution up to the present day. Among other motifs found in *env*, the motif displayed in Figure 4 was the only one for the IP –TATA box region with low *p* values between 6.09e^−18^ (SFV_spm_) and 1.02e^−9^ (Slo_EFV_). This MEME motif perfectly encompasses and extends beyond the TATA box experimentally determined for SFV/PFV, FFV and BFV, imprinted in a much larger highly conserved motif [32,50,54]. Together with the functional proof of the TATA box motif for the FV types mentioned above, one could also assume the same functional activity for the rest of the analyzed FVs, even for the endogenous FV isolates. Furthermore, this *in silico* analysis leads to the speculation that the surrounding sequences of the TATA Box motif might play an at least equal important role for the initiation of transcription as the TATA Box motif itself. The Cap site is less conserved but still present in most FV species (18 of 21; not conserved in CoeEFV and, to a lesser extent, in SFV_agm_ and SFV_mac_ (G to C exchanges)) (see Figure 4). In line with these results we set out to identify the major TATA Box that should reside within the U3 region of all 5' LTR (see Chapter 3.2) via bioinformatics. 

**Figure 5:**
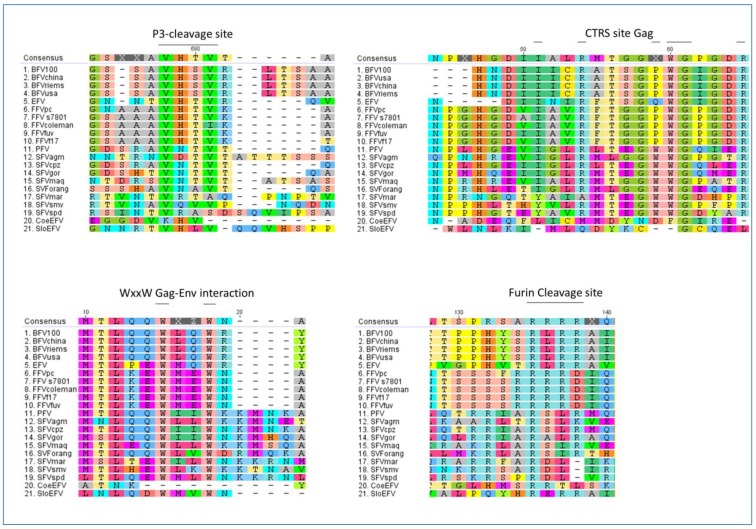
Identification of molecular features thought to be FV-specific in distinct FV species: all FVs sequences used are listed in Table 2. Here, alignments of Gag and Env amino acid sequences (in single-letter code) are shown displaying the p3 cleavage site and CTRS motif in Gag as well as the WxxWmotif in Elp and the furin cleavage site in Env. The consensus amino acid sequence is listed at the top of each set with colored capital letters. Dashes indicate amino acids missing in the isolates. The putative motifs mentioned above are indicated by bold lines over the consensus sequence. Viral abbreviations are given in Table 2. The alignment was performed with a global alignment method with free ends and gaps by Needleman and Wunsch, using a Blosum62 cost matrix [44,60,61].

Moreover, further molecular motif analyses of all FVs with completely sequenced genomes (listed in Table 2) were performed to determine whether more features, considered to be FV-specific, are in fact conserved in all FVs clades and types or lack in some of the phylogenetic branches of the computed FV phylogenetic tree. We analyzed the p3 cleavage site and the cytoplasmic targeting retention signal site (CTRS) in Gag, the tryptophan WxxW motif in Elp, required for Gag interaction and budding, and the Elp furin cleavage site in Env (Figure 5) [55,56,57,58,59]. 

The p3 cleavage site resides within the carboxy-terminal part of Gag and clips off a 3–4 kDa C-terminal peptide from the full-length precursor Gag (see Chapter 3.1). The p3 cleavage motif RA**V**N**↓**T**V**TQ is conserved nearly throughout all FV-types. All FVs, including CoeEFV with a slightly shifted motif, display a highly related potential proteolytic motif for the p3 cleavage site. Interestingly, the New World monkey FVs form the sole exception of the rule and show a partial duplication or shift of this motif.

The other important motif for particle assembly is the CTRS site within Gag. Here, our sequence comparison revealed high homologies within each FV clade. However, no conservation of the motif was found for the endogenous FVs. Either the motif underwent various mutations the endogenous FVs might have acquired or the implication is that the CTRS signal is a more recent achievement of FV evolution for more efficient capsid assembly or site specific capsid assembly. 

The FV-specific and FV-characteristic Gag-Env interaction site located in the N-terminus of Env (WxxW motif in Elp), however, is highly conserved throughout all FVs apart from CoeEFV, implicating that the Gag-Env interaction by the WxxW motif in Elp is an ancient trait of FVs as it is not only essential for Env targeting to the ER but also vital for budding (see Chapter 3.1). 

In addition, according to our phylogenetic analyses the furin cleavage site in Env also displays a conserved pattern which is specific for the different FV clades. Compared to the *Orthoretroviridae*, whose signal peptidases are responsible for the signal peptide cleavage in Env, FV exclusively make use of a furin cleavage to cleave Elp from Env (see Chapter 3.1) [62]. Therefore this ancient mechanism must have been conserved for its functionality as shown in Figure 5. The minimal consensus sequence **RXXR** is highly conserved in the Old and New World simian FVs whereas in bovine, feline equine FVs and in SloEFV, the even more efficient motif **RXRR** is present. Additionally, the furin cleavage motif is duplicated for PFV and even triplicated for SFV_mac_. Those duplicated or triplicated motifs are not only composed of the **RXXR** motif but also of the animal FV furin cleavage motif **RXRR** which is rudimentary present followed by the more general and conserved **RXXR** motif. The reason for this remains to be explained.

As an overall conclusion, it can be stated that the genetic makeup is well conserved throughout the whole evolutionary development of FVs. As shown above key FV-specific features already exist in the CoeEFV and SloEFV dating back in time as far as 400 mya. Nevertheless, in CoeEFV only the p3 site was fully conserved, while the CTRS, WxxW and furin cleavage motif are lacking. Using reverse genetics it is possible to recover the furin cleavage site by mutating the given methionine (position 133) to arginine. Vice versa, the ancient SloEFV displays three conserved motifs out of the four tested ones (p3 site, WxxW motif, furin cleavage but not CTRS site in Gag). As the host of CoeEFV is a fish and all other FV hosts are mammals, differences in motifs and/or conservation of them could be attributed to the dissimilarity of the hosts and their evolution. 

It should be noted that some sequences listed in Table 2 were not directly obtained from FVs isolated from primary tissues and cells of the original host but were rather obtained from infected cell cultures of immortalized cells including tumor cells and other cell lines not from the authentic host species. The disadvantage of immortalized cells and/or tumor cells in propagating a virus is often their specific phenotype. These cells tend to suppress/lack innate immunity to bypass potential growth restrictions by cell signaling controls, innate and adaptive immunity. Therefore FVs kept on immortalized cells and tumor cells as carriers do not face any innate immunity selective pressure which may result in lower sequence conservation (see chapter host restriction). The only exceptions are FFV_PC_ and the endogenous FVs. Obtaining molecular information from viruses derived from cell culture always bring along the burden of genetic imprint. BFV_Riems_ was kept on primary, not immortalized cells from the authentic host, which might prevent an adaptation to a new host species due to the effect of an intact innate and adaptive immunity. Most FVs, however, have been amplified by passage in heterologous cell lines, and thus from conditions with less (or different) evolutionary pressure than the original hosts itself or homologous cell lines would offer. As a consequence, genetic variations might be due to mutations and the cell culture imprint. 

## 3. Molecular Biology of FV

Compared to other retroviruses FVs show several distinct features, some of which have already been briefly described above. Some of their characteristics resemble stereotypic B/D type reverse transcribing viruses, whereas others show similarities to the *Hepadnaviridae*, and some are unique [63,64]. Explanations for such a conglomerate of traits are still lacking. One possible explanation would be the hypothesis that FVs are, due to their age (more than 400 my), the origin of retroviruses and therefore show ancient attributes of retroviruses which are no longer present in more recent lines like the lentiviruses and that the *Ortho-* and *Spumaretroviridae* developed independently over a long time span [13]. Remarkable differences are the existence of an internal promoter (discussed in Chapter 2 and below), partially reversed transcribed DNA as viral genome, Gag-independent translation of Pol as well as a highly exceptional particle assembly and virion egress reflected by unique features of FV Gag and Env proteins. Most of the work was originally performed on SFV/PFV, during the last decade, however, the animal FVs started to catch up, mostly due to research on FFV and BFV [46,63,65,66].

### 3.1. Virus Structure and Morphogenesis

As early as in the late 60s, Clarke, Dermott and their co-workers started morphological characterizations on simian and bovine FVs, analyzing overall shape and constituent parts to classify them. Although the phylogenetic classification of retroviruses in its present understanding was not evident at the time morphological criteria of retroviruses including FVs are surprisingly well reflected by the novel genetic classification [67,68,69,70]. At the time it was done morphological differences between SFV, BFV and FFV were not visible (using electron microscopy). Modern techniques, however, revealed the molecular details which are responsible for a new morphological classification.

Our review focuses on two main genes/proteins namely *gag*/Gag and *env*/Env which give rise to the viral structure.

Retroviral assembly is organized by Gag-targeting and Gag-Gag interactions. FV assembly differs dramatically from that of the *Orthoretroviridae* as has been described by Rethwilm and Linial [4,63]. As opposed to orthoretrovirus Gag FV Gag is not cleaved into matrix, capsid and nucleocapsid—only a 3–4 kDa C-terminal peptide (p3/4^Gag^) is clipped off from the full-length precursor instead. The C-terminal cleavage of Gag is necessary for virus infectivity but only incompletely executed in released virus particles [55,71]. Moreover, Flügel and Pfrepper showed additional cleavage sites in PFV Gag, mainly throughout the central part of Gag but the efficiency of cleavage is not very high compared to the carboxy-terminal cleavage. The reason for the partial cleavage of the p3 site remains to be investigated. Initially it was proposed that proteolytic cleavage is linked to viral particle formation and infectivity [72,73]. PFV particles consisting of the precursor Gag only displayed aberrant changes in morphology and scale which might be explained by the larger size of the precursor Gag and the consequently altered position of the single Gag proteins [72,74]. In contrast, processed precursor Gag as sole components of the capsid did not alter the particle assembly but yielded particles with reduced infectivity. The particle assembly from processed precursor Gag only appears to be too dense and sticky for subsequent proper disassembly after infection and therefore seems to reduce infectivity [75]. Thus the carboxy-terminal processed p3/4^Gag^ might have as yet unknown functions and possibly plays an important role in the correct spacing of the capsid for subsequent full infectivity. The evolutionary conservation of the p3 cleavage site throughout all FVs underlines the importance of its function (see above).

Within the *Orthoretroviridae*, the cleavage of Gag modulates membrane tracking and budding by disclosing virus specific motifs [76]. As FVs generally do not display a typical retroviral myristylation motif and do not exhibit N-terminal basic sequence sections, a mechanism other than the mentioned must be in charge of membrane targeting. To address this issue, Wilk and co-workers picked up the track and analyzed PFV and FFV capsids by cryo electron microscopy (cEM), paired with surface plasmogen resonance (SPR) analyses [17]. It became evident only by cEM that the differences in matrix layer formation and size are characteristic for different FVs due to the genetic makeup and size of *gag*/Gag [17]. The central and C-terminal part of all FV Gags is basically identical in size and structure, thus yielding identical dimensions of the capsids. Moreover, the constant N-terminal part is responsible for the targeted Gag assembly at the microtubule organizing center (MTOC), a structured process which already might have been observed in low resolution by Clarke *et al.* in the late 60s [68,77]. Other than the central and C-terminal part of Gag, the N-terminal part (between aa150–aa350) varies in size and is mainly responsible for the differences in matrix layer dimension [17]. Moreover, the N-terminal part contains an Env interaction domain which is critical for the virion egress [17,58,77]. 

As far as Env is concerned the interaction with Gag is mediated by Elp, the Env leader peptide. Signal peptides like Elp generally have the function to target Env towards the ER for subsequent processing of Env accompanied by the degradation of the signal peptide. As opposed to the *Orthoretroviridae* FVs almost exclusively make use of a furin cleavage site to clip Elp off the SU-domain of Env [59,78]. Despite this fundamental difference of using a furin-mediated cleavage instead of signal peptidases as observed for other retroviruses FV Elp has an additional post-targeting function since it was proven for PFV and FFV, that Elp is incorporated into the virus particles and is vitally essential for viral budding, for the highly conserved tryptophans in Elp are required for the Env/Gag binding, as shown in Figure 5 [17,58,79]. Furthermore, mutation studies revealed that the secondary structure and overall sequence region also participate in the interaction process [17]. The absence of the WxxW motif in CoeEFV is obvious considering the sequence comparison in Figure 5 might be explained by the occurrence of existing mutations in the endogenous FV. Reverse sequence analysis including single nucleotide mutations and an induced frame shift would restore the tryptophan motif (data not shown). 

In addition to the conserved tryptophan motif, the Elp peptide harbors some ubiquitination sites at K_14_, K_15_, K_18_, K_34_ and K_53_ which have been functionally analyzed in PFV [80]. Detailed analyses revealed that combined ubiquitination of only the first three sites effectively blocks the release of subviral particles (SVP), while high SVP release is observed upon mutation of these sites. Besides the well-known degradation process induction ubiquitination in PFV therefore exhibits a second function in the context of viral egress and infectivity. More recent analysis by the same group revealed that only the first two lysine residues are important with regard to ubiquitination and suppress SVP release to wild type levels [81]. As FV-Env is, in contrast to all other retroviruses capable of budding independent of any other FV protein, this mechanism might tightly regulate SVP release, at least in PFV.

Sequence comparison reveals some conservation of the lysine residues evident in PFV among the other SFVs. Moreover, Stanke and coworkers demonstrated ubiquitination of Env LP in PFV particles pseudotyped by SFV_mac_, suggesting that this post-transcriptional modification is not unique to PFV and might be common to all FV species [80]. Nevertheless, ubiquitination seems not to happen in FFV (unpublished data from the Löchelt lab, personal communication with Anne Bleiholder, Martin Löchelt, Peter Wirthschaft) [82].

With respect to budding, additional differences can be observed between PFV and the animal FVs: PFV tends to bud preferentially from membranes of the ER and rather rarely from the plasma membrane which might be due to the dilysine motif in the cytoplasmic tail of the TM glycoprotein of PFV [83]. The dilysine motif is composed of two lysine residues within the last 5 aas with respect to the carboxy-terminal end of the TM part of Env [84,85]. In PFV mutations within this motif enhance budding from the plasma membrane, though the subsequent production of extracellular infectious viruses does not seem to raise significantly [84]. The dilysine motif has also been described for SFVs and BFV [86], though for BFV the motif is not fully preserved and appears to be non-functional as BFV buds exclusively from the plasma membrane [86,87]. These new findings from Kong and co-workers are contrary to old thin section EM analyses from Dermott *et al.* which showed budding into intracellular membrane compartments [69]. The observation of Dermott and coworkers would be in line with the hypothesis that the lack of lysine in critical positions -4 or -5 (with respect to the carboxy-terminal part of TM) could be compensated by an arginine restoring the ER-retrieval signal [85,86]. The EFV Env glycoprotein does not harbor the dilysine motif at all and was experimentally proven to bud exclusively from the plasma membrane [33,88]. FFVs exhibit an ER dilysine retrieval signal, **KK**DQ which closely resembles that of SFVs. In line with that, budding is observed at the plasma membrane but also at intracellular compartments [89,90]. All in all it seems unclear why FVs should have established such an ER dilysine retrieval signal.

The conservation of this signal varies among the different FV clades resulting in a continuous spectrum of budding strategies among them whose outer ends budding primarily from the ER and budding primarily from the plasma membrane—are represented by PFV and EFV respectively. Combinatory budding strategies seem to be preferred by BFV and FFV which are capable of using both. Therefore it seems conceivable that the ER dilysine retrieval signal might have some additional functions like the Gag/Env assembly.

In summary, morphological analyses of FVs reflect in part the differences revealed by the phylogenetic and molecular analyses showing rather distinct FV clades instead of a single homogenous FV family. Morphogenetic analyses, for instance, confirm the conservation of the p3 cleavage site in Gag and demonstrate the consequences of mutations within this highly conserved site. Furthermore, the different sizes of the N-terminal Gag region in primate and animal FV could be linked to different dimensions of the matrix layer in PFV and FFV as seen in cEM analyses. The differences in ubiquitination between PFV and FFV and the subsequent consequences for SVP release became visible, but the biological implication of this feature needs further validation for the different FV types. Moreover, morphological analyses revealed differences among FVs in their budding strategy indicating a possible FV clade-specific host dependency or a mechanism of viral egress regulation and/or avoidance of immune recognition in the different hosts. 

### 3.2. Foamy Viral Transcription

It seems very likely that only upon integration into the host DNA the FV provirus DNA genome is transcribed into viral RNA. Transcription is carried out by the host cell machinery, *i.e.*, cellular RNA polymerase II with associated host enhancer binding proteins. With the exception of the transcriptional termination signal, the transcriptional start control elements of FVs are located in the U3 region of the 5' LTR and upstream of the internal promoter at the 3' end of *env* which is a unique feature of FVs distinguishing them from all other retroviruses [4,63]. To our current knowledge the transcriptional control units of FVs consist of many different elements, among them one or more Tas response elements (TREs), the TATA box and the downstream located cap site. As transcriptional transactivator FVs express Tas (see Figure 1) which binds with high affinity to the TRE in the internal promoter site and initiates transcription of its own sequence in a positive feedback loop manner and Bet, respectively [91,92]. Once a certain critical concentration of Tas is reached, Tas most likely also binds to additional TRE(s) in the U3 region of the LTR of FVs and initiates transcription of *gag*, *pol*, and *env*. Therefore, FV replication might be regulated by mechanisms that involve a temporal pattern of gene expression. The higher affinity of Tas to the IP and higher basal transcriptional activity compared to the LTR promoter may explain this bimodal temporal pattern [51,52,93,94]. At the earliest stage of gene expression when FV Tas is not expressed yet the basal IP transcriptional capacity depends on host cellular factors and also on the provirus integration site, but the potential cellular factors involved and the precise mechanism of how IP transcription is activated remains to be elucidated. 

Although the LTR-TREs of PFV, SFV_mac_, SFV_agm_, BFV and FFV have all been mapped to the viral LTR U3 region, the number and position of the LTR-TREs are not conserved among the FVs. For instance, PFV LTR contains three TREs located at the positions -360/-342, -327/-284 and -116/-89 relative to the transcriptional start site [95]; SFV_mac_ LTR contains two TREs (-1196/-880 and -403/-125) [96]. Furthermore, two segments (-637/-496 and -496/-180) have been mapped to the SFV_agm_ LTR [97]. In the FFV LTR two regions with the same function have been mapped to -228/-195 and -66/-51 [98]. A more precise analysis of TREs in BFV revealed that two segments (-983/-668 and -380/-140) in the LTR respond to BFV Tas; however, the region at -380/-140 was more important for transactivation by BFV Tas than that at -983/-668 [99]. Unlike other FVs, the BFV TREs exhibit sequence homologies, and four conserved elements (-368/-346, -327/-307, -306/-285, and -186/-165) in the BFV LTR. Moreover, the TRE element (-380/-140) was found to be crucial for the binding of BFV Tas to its target sites [92]. However, to our knowledge the sequence does not show obvious homologies to any other known eukaryotic enhancer sequence motif. In addition, there is no obvious sequence conservation among the TRE of different FVs. This may indicate the co-evolution of FVs with the host, resulting in different Tas proteins and TREs.

Similar to the LTR-TRE the minimal IP-TRE sequences were mapped in PFV (-166/-140) [100], SFV_mac_ (-69/-44) [101], FFV (-70/-58) [102] and BFV (-34/-13) [92]. It could be shown that the IP of PFV and SFV_mac_ to a certain degree display sequence similarities. Apart from that this IP-TRE does not demonstrate any marked identity.

**Figure 6:**
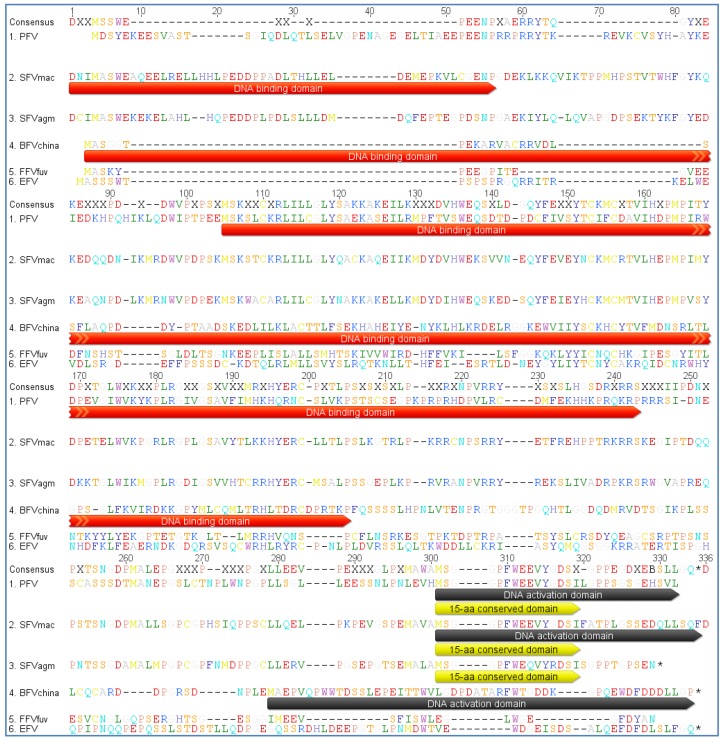
Protein sequence alignment of Tas sequences from PFV, SFV_mac_, SFV_agm_, BFV_China_, FFV_FUV_ and EFV (see Table 2 for accession numbers). The consensus amino acid sequence is listed at the top of the alignment with a 0% Majority argument, which lists only the most common bases with colored capital letters. Dashes indicate amino acids missing in one or more isolates. The amino acids are displayed in a single letter code in Rasmol style. The DNA binding domain (DNA-BD), the activation domain (AD) and the 15 aa conserved sequence motif are indicated by a colored shading in the sequences (DNA binding domain = red; DNA activation domain = grey; 15aa conserved domain = yellow). The alignment was performed as a global alignment with free ends and gaps by Needleman and Wunsch, using a Blosum62 cost matrix [44,60,61].

The sizes of Tas proteins encoded by PFV (300 aa), SFV_mac_ (308 aa), SFV_agm_ (301 aa), BFV (249 aa) and FFV (209 aa) are highly divergent, and the sequence comparisons of FV Tas reveal a low level of conservation [24,28,103,104]. However, in all FV types Tas has equivalent transactivation function and consists of at least two functional domains, a DNA-binding domain (DNA-BD) and an activation domain (AD) (see Figure 6) [92,105,106]. A 15-aa conserved sequence motif is located in the AD of the three Tas proteins of PFV, SFV_mac_ and SFV_agm_, but this motif is not found in Tas of BFV and FFV [92,106]. However, the AD of FV Tas belongs to the class of acidic transcriptional transactivators [92,106,107,108]. As distinct to PFV Tas the deletion of 15 amino acids at the C-terminus of BFV Tas severely diminished the activation ability of the transactivator. Aside from these two domains BFV Tas also contains one negative regulatory domain similar to PFV Tas [109].

Dimerization of transcription factors and activators is a common phenomenon associated with gene transcription. It was reported that PFV and BFV Tas proteins form dimers in the nuclei of mammalian cells [110,111,112]. In contrast to BFV Tas, PFV Tas has three domains that mediate dimer formation and the comparison of the dimerization domains of both proteins did not reveal obvious homologies [111]. In order to be functional dimeric BFV Tas has to bind to the DNA response elements [110]. It is possible that these protein-protein interactions of fully assembled dimeric BFV Tas on the DNA response elements are essential for further interactions with cellular cofactors needed for BFV Tas function. However, the biological function of PFV Tas dimerization also needs to be defined. 

Recent investigations indicate that p300 and PCAF specifically interact *in vivo* with PFV Tas protein resulting in the enhancement of Tas-dependent transcriptional activation [113]. Subsequently FFV Tas was proposed to be acetylated by PCAF leading to increased promoter-binding ability [114]. Similar to FFV Tas the p300 acetylation of BFV Tas can increase its DNA binding affinity, and the K66, K109 and K110 are critical residues for the DNA binding ability of BFV Tas [115]. These findings suggest that acetylation is a ubiquitous mechanism adopted by FVs as a way of regulating gene expression and that animal FVs potentially share similarities with PFV in their need for essential host cell factors, e.g., p300 and PACF, *etc.*

**Figure 7:**
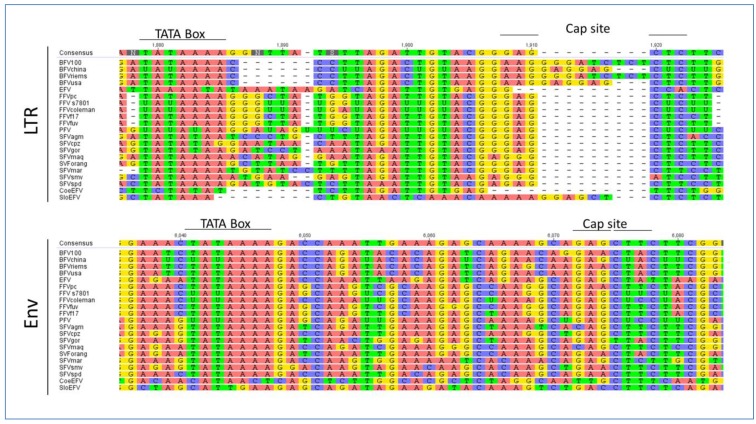
Multiple genomic sequence alignment of the TATA box motifs in FVs: All FVs sequences used are displayed in Table 2. As sequences have been selected according to the GenBank entries, some FV genomes are listed as RNA instead of DNA genomes, therefore containing uracil (U) instead of thymin (T). Alignments have been done for the LTR and *env* region. The consensus nucleotide sequence is listed at the top of each set with colored capital letters. Dashes indicate nucleotides missing in the isolates. The TATA box motifs are indicated by bold lines over the consensus sequence. The alignment was performed with a global alignment strategy with free ends and gaps by Needleman and Wunsch, using a Blosum62 cost matrix [44,60,61].

Regardless of the size and location of the TREs and the sequence of Tas the latter will facilitate interaction with transcription factors and other factors from the whole transcription machinery. As already described above, using the MEME motif search program we identified a conserved motif including the TATA box in *env* in all FVs. Additionally, we analyzed the U3 region of the LTR of all FVs as listed in Table 2. We identified a TATA Box similar to the one in the *env* gene but the overall surrounding sites are less conserved (Figure 7). Our findings are in line with the fact that the LTR is a highly variable region often affected by mutations and recombination events in contrast to the *env* IP region, which resides in the important and conserved *env* gene possibly imposing additional selective pressure for primary nucleotide sequence conservation.

EFV shows a positional shift in the TATA Box location, while most of the Old World SFVs and also the CoeEFV display an alternative TATA Box motif which is, slightly modified compared to the typical consensus sequence **TATAAA**, but obviously still valid. The Cap site and the surrounding sequences are also conserved throughout the different FV types, though interrupted by an insertion in the BFV species and in the SloEFV (see Figure 7). Nevertheless, this insertion is equaled by the absence of a sequence cluster in BFV, compared to the other FVs, indicating that the overall TATA box motif appearance is intact. The insertion in the SloEFV might be explained as for BFV, but one has to consider that endogenous elements are subject to a higher mutation rate which might also be an explanation for this insertion. 

Taken together these data point towards structural differences between the different FV species in Tas as well as in TRE and TATA box elements of LTR and *env* whereas the overall functional seems undisturbed. Within a FV clade, the elements are well conserved and the differences in the LTR and *env* promoters may be the reason for the discriminative gene expression in these transcriptional units. Finally one has to consider the interaction of Tas with cellular components involved in transcription and gene expression. Tas of PFV has been shown to activate a substantial number of cellular genes maybe also leading to their dysregulation [116]. This poorly understood issue still needs to be solved to understand the corresponding cellular processes and to deepen the risk assessment for FV vector design and application.

### 3.3. Host Restriction

The successful and latent infection of various hosts by retroviruses can be explained by the ability of the latter to bypass cellular counteraction mechanisms by means of limiting viral replication. Whilst the virus makes use of supportive host factors to facilitate its replication, inhibitory factors of the host try to counteract viral replication [117]. These inhibitory factors are called host restriction factors and potentiate the early block in viral replication alongside the first line of defense (the innate immunity) [118,119]. As viruses evolve very fast, the host also has to co-adapt to balance the race [118,120]. Prominent examples are lentiviruses, in particular the zoonotic transmission of SIV to humans in the last century, eventually yielding in HIV type 1 and 2. Zhang and co-workers showed that the co-evolution of primate SAMHD1 (SAM domain and HD domain-containing protein 1) and lentiviral Vpx leads to the loss of the *vpx* gene in SIV_cpz_ (Simian Immunodeficiency Virus) and HIV-1 [121]. 

In the course of time, we significantly enhanced the knowledge about host restriction factors and several restriction factors have been identified so far, among them the most prominent include Fv1 (Friend virus susceptibility factor-1), TRIM5α (tripartite motif 5), Tetherin (BST-2; bone marrow stromal antigen 2), SAMHD1, and APOBEC3s (apolipoprotein B mRNA-editing catalytic polypeptides) [118,122,123,124,125,126,127,128]. Different members of the retrovirus family use different strategies to overcome these host restriction factors. As *Spumaretroviridae* display remarkable differences in the viral life cycle, knowledge of host restriction factors gained from lentiviruses may not be transposed directly to FVs. So far little is known about FV counteraction of host restriction factors like SAMHD1 or Fv1, but for some of the listed factors initial discoveries have been made: (1) Yap *et al.* showed restriction of foamy viruses by primate Trim5α targeting FV Gag which holds true for PFV, SFV and FFV as well [129]. Nevertheless, as discussed by the authors, this study failed to show a clear protective role for Trim5α, since Trim5α from New World simians was able to block SFV and PFV but not FFV; vice versa Old World monkey Trim5α was able to block FFV [129]; (2) In contrast, Pacheco *et al.* demonstrated the ability of Trim5α to restrict SFVs in New World Monkeys. They could show that specific Trim5α proteins from New World monkeys are able to diminish the replication of certain SFVs indigenous to the New World [23]; (3) Xu *et al.* showed a possible restriction of PFV by Tetherin and therefore the inhibition of infectious PFV particle release [130]; (4) Jouvenet and coworkers confirmed the findings from Xu *et al.* [131]. Moreover, they demonstrated with modified PFV Gag proteins (Lck-Gag) that the activity of Tetherin was largely independent of the mechanism by which Gag was targeted to the membrane. The main conclusion of this large retroviral and filoviral study is that Tetherin acts independent of viral protein sequence identity as a “broad-spectrum” inhibitor, possibly by cross-linking the viral and host lipid bilayers after budding [131].

Compared to those preliminary studies there is profound knowledge by many research groups that the FV accessory protein Bet counteracts viral host restriction imposed by particular APOBEC3 (A3) cytidine deaminase restriction factors [132,133,134,135]. Nevertheless, Delebecque’s work using SFVs and APOBEC3 from murine, simian and human hosts does not support the work of Löchelt, Russell, Munk and many more, which might be due to the variable expression levels of Bet and APOBEC3s used or the different experimental setup. [132,133,134,135]. A3 proteins are members of the AID/APOBEC (activation-induced cytidine deaminase) protein family and counteract viral replication by deamination of cytidine residues in single stranded DNA molecules as part of the reverse transcription process [123,136,137]. In addition it was proven that A3s are also capable to restrict non retro-transcribing viruses like human papilloma virus (HPV) [138]. In order to counteract retroviral replication APOBECs are incorporated into the viral particles and this incorporation might be facilitated by the viral Gag protein, as an interaction and co-localization was shown by several research groups [139,140,141]. Comparable to the Vif counteraction of A3 in HIV FVs may have acquired Bet to counteract A3, though the mechanism of counteraction is fundamentally different (see below).

Bet proteins of all known FVs are derived from a spliced mRNA which fuses the 5' part of *tas* to the complete *bel2* ORF. Differences between the different FV species in the molecular makeup of Bet have already been discussed elsewhere [1]. So far, the three dimensional structure of Bet is still unknown. Therefore detailed knowledge of the binding site of Bet and A3 and the detailed mechanisms of interaction between Bet and A3 are also missing. Based on one study and the observation of possible homo and heterodimers of A3s it seems conceivable that Bet might bind to this dimerization domain thus inhibiting A3 [142]. The analogous protein Vif in HIV on the other hand counteracts A3 via degradation [128,143,144]. FFV Bet proteins bind A3s but do not induce their degradation. Moreover, a mechanism was proposed where Bet blocks the package of A3 into the new virions [132,133,134,140]. This would be in line with observations showing Bet being highly overexpressed in the host cell possibly to efficiently block A3 in a ratio dependent manner [145]. Due to the coevolution of restriction factor and viral defense factors FFV Bet is capable to block feline A3s but not simian or human A3s in a highly efficient manner, while SFV/PFV Bets are able to inhibit simian and human A3, concluding that viral counteraction is only possible in similar species.

In summary, we can say that animal FV research has had significant impact on viral restriction knowledge so far. In particular, research performed with FFV Bet contributed to the deeper understanding of the Bet-A3 counteraction. Due to the lower number of A3 genes within the *Felidae*, (so far there seem to be four), the FFV Bet/APOBEC3 interaction studies are superior to the human related PFV studies which have to consider seven different A3 genes. Therefore, using FFV with their authentic hosts might be a more comprehensive approach to conduct animal experiments for restriction analyses. The coevolution of Bet and A3 must have been going on for a long period of time, its origins possibly dating back up to 100 mya culminating in this highly specific interaction process to counteract viral restriction, as FV Bet sequence identity was even found for the SloEFV [12,134,146]. Elucidation of the three-dimensional structures and therefore the binding site(s) of Bet and A3 will give rise to further investigations to deepen our knowledge of the virus-host restriction arms race. 

## 4. Biology in the Host

### 4.1. FV Tropism

**Figure 8:**
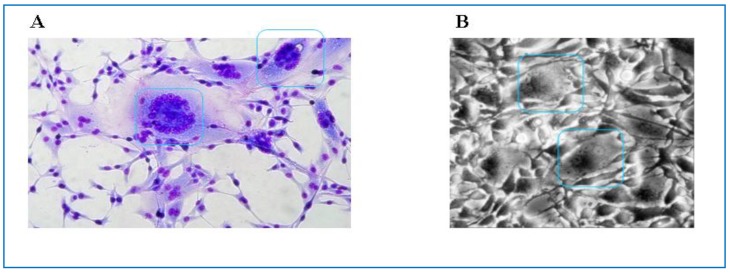
(**A**) Characteristic cytopathic effect observed in BFV-infected canine thymus cells (Cf2Th). Blue boxes frame multinucleated cell fusions (syncytia formation) which are characteristic for BFV-infected Cf2Th cells. Cells have been Giemsa stained; (**B**) Syncytia formation in FFV infected Crandell feline kidney (CRFK) cells, as seen by phase contrast microscopy. Blue boxes indicate cell fusions with multiple nuclei (by courtesy of Anne Bleiholder for (**B**)).

It is well known that FVs have the capacity to infect diverse cell types of vertebrate origin from fish to humans. Most of these data originate from studies on PFV and SFVs [147,148], animal FVs, however, are able to grow in cell lines or primary cells of different origin as well [19,149]. In most of these cell types FV infections are highly cytopathogenic (Figure 8). In fibroblasts or fibroblast-derived cell lines as well as in many epithelial cells infection with animal FVs leads to rapid death of the cells preceded by formation of highly vacuolated multinucleate syncytia with foamy appearance as has been shown by electron microscopy [13,16,150,151]. Interestingly, in contrast to primate FVs animal FVs seem to cause lytic infections mainly *in vitro*, but persistently infected cells can be established *in vitro* without cell death [152,153]. *In vitro* infection with all types of FVs is highly cell associated causing problems with virus harvesting and titration especially in the case of animal FVs. Therefore special indicatory cell lines like BICL cells (BFV indicator cell line) for BFV and FeFAB cells (Feline Foamy Virus activated β-Galactosidase expression cell line) for FFV have been developed similar to FAB cells (Foamy Virus activated β-Galactosidase expression cell line) for PFV [149,154,155]. 

As with other retroviruses, *in vivo* tropism of FVs to blood cells was investigated. Some reports suggested that CD8^+^ T cells may be the SFV reservoir in simians, but this could not be confirmed by another study in which only monocytes and B cells were found to harbour FV viral DNA, so that the issue still remains disputable [156,157]. Some limited *in vivo* studies on animal FVs performed so far showed quite wide tissue tropism of FFV and BFV in natural hosts and for BFV even in BFV heterologous hosts [158,159]. In some of the reported studies BFV, FFV and EFV were recovered simply from peripheral blood leukocytes/lymphocytes but FFV and BFV were also recovered from tumors and BFV itself from fetal tissues, placenta and testis as well as from fluids used to flush the uterus and oviducts of super-ovulated cows [30,33,151,153,160,161,162,163,164,165]. Interestingly, recent studies on naturally or experimentally infected animals using polymerase chain reaction (PCR) technique show that FFV and BFV DNA is present in most tissues (*i.e*., lung, salivary glands, liver, spleen, bone marrow) [3,158,159,166]. However, so far there are no data regarding the presence of FFV or BFV RNA and a possible replication of animal FVs in those organs. Some previous reports from SFV studies in monkeys showed that although SFV DNA is present in most animal tissues, SFV RNA, indicative of viral gene expression and replication, is primarily detected in oropharyngeal sites [166,167]. Therefore we can assume that the mentioned organs are not the primary target of FVs and detection of FVs DNA is rather due to their high vascularization and an enrichment of infected leukocytes [142]. 

So far, no detailed studies on potential sites of virus replication were undertaken for animal FVs. For our own studies BFV was isolated from saliva and milk cells [145,168]. The presence of infectious BFV particles in those samples seems to be strong proof for the hypothesis that the active replication may not be limited to the oral cavity only, as previously suggested for monkeys but can also be located at other sites, for example in udder [166,167]. Furthermore, it can be presumed that in addition to saliva, milk may be another route of FV transmission.

### 4.2. FVs Prevalence

In contrast to some well-studied SFVs which were investigated in captive as well as in free-ranging monkeys and apes, prevalence of animal FVs has been much less thoroughly studied. Available reports from Europe, Australia and Asia showed high FFV prevalence in domestic cats ranging from about 30% to even 100% depending on sex, age and geographical region analyzed [169,170,171,172,173]. FFV-like viruses were also found in wild felids and are detectable in 35% of an endemic cat species from the Japanese island Iriomote (*Felis Iriomotensis*) and in one leopard cat species (*Felis bengalensis*) from Vietnam [34,174]. Interestingly, our sero-epidemiological data suggest considerable presence of FFV-like viruses in free-ranging North American pumas (*Puma concolor*) and bobcats (*Lynx rufus*) from different geographic regions in the US as analyzed by serology using FFV antigens (personal communication with Anne Bleiholder and Martin Löchelt) [175,176]. BFV infections among cattle were reported worldwide and ranged between 30% to 45% [177,178,179]. In other studies the BFV seroprevalence was estimated at 7% and 30%–40% in dairy cows from Germany and Poland, respectively (unpublished data from the Kuzmak lab) [180,181]. In addition to the epidemiological studies on BFV, it was demonstrated that domestic sheep across Germany showed a seroconversion for BFV antigens (Gag, Bet and Env) (unpublished serological data from the Löchelt and the Kuzmak lab) [90,181]. Follow-up studies should be able to prove whether this is due to a unique, highly BFV related, FV in sheep, or an interspecies transmission of BFV to sheep [19]. Up to this day, the prevalence of EFV is unknown, but preliminary studies on saddle horses, Hucul ponies and semi-feral Polish primitive horses showed the presence of provirus nucleic acid of EFV in about 15% of the tested animals [182]. 

### 4.3. Viral Transmission

Despite many studies on FVs, detailed information about FV transmission is still lacking. The extraordinary wide host range of FV *in vitro* is thought to be the consequence of a very widespread viral entry receptor present on the cell surface. Recent studies even suggest heparan sulfate proteoglycans as receptor or attachment factor for FV uptake [183,184]. In line with this, our studies on FFV and BFV demonstrated that the virus in infected animals is present in nearly every analyzed tissue. Anyway, since oral mucosa is considered to be the main site of FV replication *in vivo*, saliva appears to be the basic route of FV transmission [173]. In primates SFV is presumed to be mainly shed through severe bites bringing infectious saliva in contact with blood [3,185]. For FFV transmission on the other hand, rather non-aggressive behavior such as intimate social contact enabling salivary transfer of FFV between animals, seems to be of particular significance [173,186]. This appears to be also possible for BFV transmission, since among cattle aggressive behavior is rather less common. Therefore it is presumed that BFV shedding occurs via saliva through close non-aggressive contact (sneezing or licking) which could explain the situation reported by Johnson and others who showed that calves BFV negative at birth became infected within 3 years when kept together with infected adults [178]. In this context also feces can be presumed source of infection for cattle, as it was suggested for SFV in chimpanzees [181]. Additionally, perinatal modes of transmission have also been proposed via colostrum or milk, for instance. This route of transmission is supported by studies showing that BFV can be reproducibly isolated from raw milk cells and therefore may have significant implications for interspecies or zoonotic transmission of BFV [168,180].

### 4.4. Interspecies Transmission

The notion of FVs being transmitted between species is quite obvious since they are widely spread among different species, have a highly conserved genetic makeup and are commonly transmitted via saliva. In fact cross-species transmission of monkey SFVs was already confirmed in wild-living chimpanzees and most likely resulted from their predatory nature [187,188]. Moreover, those studies demonstrated that animals carrying their own species specific SFV can be infected with divergent SFV strains, indicating that FVs are prone to superinfection [186,188,189]. Such findings suggest the possibility of recombination which could have implications for foamy viruses as vaccine vectors and their potential pathological properties [187,190]. First indications for a potential pathological role of FVs were published by Murray *et al.* and Choudhary *et al.* Murray *et al.* showed an enlarged tissue tropism for FV replication in double infected (SIV and SFV) simians. The dysregulation of the immune system by SIV leads to this expanded tissue tropism of SFV [167]. More recently, Choudhary *et al.* demonstrated that SFVs exhibit a cofactorial role in SIV infected simians and alters the disease outcome in the used rhesus macaque model [191]. 

Similar to SFV, animal FVs could also be able to undergo interspecies transmission. Limited reports have suggested the possibility of FFV or BFV transmissions to other closely related feline or ungulate species. FVs were evidenced in different species of wild cats and bisons by virus isolation, detection of specific antibodies by immunofluorescence assay (IFA), enzyme-linked immunosorbent assay (ELISA) or by positive amplification of FV DNA and in most cases their homology to FVs was additionally confirmed by sequencing [34,173,174,192,193]. Surprisingly, all of the mentioned reports show that FFV- or BFV-related viruses were frequently found in wild animals while other retroviruses, *i.e.*, feline immunodeficiency virus (FIV), bovine immunodeficiency virus (BIV) or bovine leukemia virus (BLV) were undetectable or clearly less frequent in feral cats as described by Winkler and coworkers (36% seropositive FFV cats *vs.* 9% FIV seropositive cats) [173,174,192,193]. Investigations of the genetic diversity and phylogenetic relationship among FFV isolates from domestic cats and FFV‑related viruses from wild felids of geographically distinct areas in Japan (Iriomote cats and leopard cats) showed that Iriomote cat isolates were genetically closer to the FFV isolated from domestic cats than those from the leopard cats [34]. Interestingly, Iriomote cats inhabit Iriomote Island which is located off the south-coast of Japan and was geographically isolated about 200,000 years ago, therefore the possibility of FFV transmission between domestic and Iriomote cats, suggested by Phung, is very likely [34]. As already stated above, high identity between FFV of domestic cats and the one recently found in pumas may either be linked to an interspecies transmission of FFV from domestic to wild cats or to the same evolutionary pressure both species are exposed to. Similarly, the clear antigenic relationship of an American bison isolate and BFVs may suggest transmission of the virus from cattle to wild bovines, although independent coevolution of those infections in two bovid species also appears to be possible [192]. Recently, 300 wild ruminants including red deer, roe deer, European bisons and fallow deer were tested, and seroreactivity to BFV antigens was found in about 5% of animals. However, only one DNA sample isolated from red deer blood cells reacted in PCR with BFV *pol* gene specific primers and the resulting amplicon showed 93% identity in comparison to BFV_USA_ isolates (GenBank Acc no. U94514; also see Table 2) [168]. These results suggest either a possible contact with BFV through shared grazing areas creating a risk of BFV transmission to wild ruminants or indicate the presence of divergent FVs, specific for those ruminants. This might also be the case in our ongoing study on sheep and goats which revealed reactivity to BFV antigens in over 20% of serum samples (unpublished data from the Löchelt lab) [90]. Such seroreactivity could be due to contact with BFV infected cows, however, the percentage of seropositive samples indicates rather that we might have found a divergent small ruminant specific FV as was previously reported by Flanagan [19]. So far these questions cannot be answered yet due to the lack of any verification, for instance by virus isolation. 

Obviously, further studies are required to provide new insights into the potential origin of wild cat and wild ruminant FVs as well as other novel exogenous and endogenous FVs. So far there were no reports involving interspecies transmission of EFV. 

### 4.5. Zoonotic Potential

Due to the zoonotic potential of SIV, the transmission of SFV to humans raised many public health questions since it was reported in persons occupationally exposed to contact with monkeys and apes, as well as people having contact with primates in natural settings of Africa and Asia [47,194,195,196,197]. The major risk factor for virus acquisition seems to be severe bites from adult non-human primates (NHP) but some reports indicating SFV transmission through percutaneous and muco-cutaneous exposures to NHP body fluids imply that also butchering or simply direct contact by working with animals may be hazardous [9,47,198]. 

Similar to SFV, animal FVs may pose zoonotic threat, too. The risk of animal to human transmission arises either by contacting BFV, FFV and EFV infected animals or by food and medical products derived from FV infected hosts [46]. So far, two studies involving people occupationally exposed to FFV infected cats did not provide evidence for any signs of potential zoonotic transmission [199,200]. Surprisingly, in our own studies enrolling veterinarians, dairy cows’ caretakers as well as cattle owners, we showed seroreactivity to BFV antigens in over 7% of the samples. Since we did not detect viral DNA we can only state that those people indeed had contact with the virus, the issue of virus transmission, however, remains disputable and should be further analyzed by independent assays [168]. Moreover, we should be aware of the fact that the risk of BFV exposure through routes other than direct contact like for example the consumption of raw milk or butchering associated with the possibility of skin injuries or aerosol production and inhalation, may increase the risk of BFV transmission to humans [1]. Nothing is known about zoonotic transmission of EFV, however, by analogy to FFV and BFV it can be expected to occur not only by direct contact with horses but also by butchering and meat consumption, however, this has not been studied or proven yet. [46]. On the other hand, one can assume that the risk of animal FV zoonotic transmission to humans is significantly lower than that of SFV, since compared to primates, cats, cows and horses are clearly less genetically related to humans. Thus virus entry can be simply stopped by host-determined adaptive, humoral and cellular immune mechanisms as well as by innate immunity. However, it is likely that such barriers for virus transmission may be limited in immuno-compromised individuals or even in children with a developing immune system [46]. Therefore these groups of humans should be considered as highly exposed to the risk of animal FV transmission, especially those having direct contact to cats, cows and horses.

## 5. FV Diagnostic Methods

In general, in the absence of a vaccine or possible therapy, an early diagnosis for viral infection is considered indispensable to prevent any harm. As FVs are believed to be apathogenic which indeed still needs to be proven the focus of detection and diagnosis rests rather on transmission events, viral load, screening possibilities and its application for vector development than on the prevention of any casualty. Therefore, in the past decade various methods were developed to screen for FV antigens, nucleic acids and viral particles. In contrast to observation of viral particles by electron microscopy, modern immunological and molecular methods could be used for high throughput diagnostics. 

### 5.1. Immunological Methods

In many hosts where FVs are prevalent, serum antibodies as well as neutralizing antibodies against FV proteins have been associated with FV infection [201,202]. Currently, for nearly every exogenous FV species serological tests are available. These include immunofluorescence, immunoblotting and ELISA based assays. For SFV, a variety of different immunological assays have been developed so far. Most of these assays address the viral proteins Gag and Bet. In ELISA and immunoblotting, positivity is determined by the reactivity against the Gag precursor proteins p71/p68 [145,180]. With regard to detection and screening, the animal FV research field does not fall too far behind and almost reaches the same standard. Jacobs *et al.* started in 1995 with sero-epidemiological analysis of cattle in Ontario, USA [177]. By now, a variety of serological analyses has been described for FFV and BFV [20,145,175,203]. Going one step further, a serological analysis was set up for livestock cattle shedding BFV into milk, making diagnostic methods even non-invasive compared to classical immunological analysis [180]. Taking advantage of the partial cross-reactivity of FV antigens, it is also possible to detect other unknown but antigenetically related FV species, as our group could show a BFV like FV prevalent in sheep and a FFV-like FV in pumas and bobcats (unpublished data from the Löchelt lab) [90,204]. These methods offer the chance to complete the list of hosts susceptible for FV infections. 

### 5.2. Molecular Methods

Molecular diagnostics of FVs is based on PCR methods and allows detection of viral DNA in blood leukocytes and other tissues. PCR detection of primate FVs DNA is frequently based on primers designed to match part of the *pol* gene at the boundary next to the RNaseH domain of reverse transcriptase and integrase [205,206,207]. This region was recognized as one of the most highly conserved sequences in the FV genome. In contrast to the detection of primate FV infections, the identification of FFV DNA based on primers matching *gag-pol*, LTR and *tas* regions [34]. Most of those classical PCR based methods were used for genotyping but were frequently also implemented for routine diagnostics. In another study German and others introduced FFV specific quantitative PCR (qPCR) which amplified a 111 bp fragment of *pol* gene and this method was successfully used for the estimation of FFV load in tissues of experimentally infected cats [158]. 

BFV diagnostic PCR was initially developed by Pamba and others and enrolled two independent PCR methods with primers matching the *gag* and the *pol/env* region of the BFV genome [179]. PCR specific for the *pol/env* region turned out to be particularly sensitive for BFV detection in DNA from cell-culture or lymphocytes of infected animals. Since both methods based on conventional PCR methodology, the sensitivity level was not satisfying, however, it could be increased by Southern blot hybridization. Higher sensitivity was achieved by Lew and others who designed qPCR technique with TaqMan^®^MGB probe matching the same *pol/env* region and detected BFV DNA in as little as 30 pg of DNA from infected cells [208]. Independently, nested PCR and qPCR with Sybr Green chemistry were developed for BFV DNA detection and quantification in field samples as well as in animal tissues [209]. All primers used for these amplifications matched the *pol* gene fragment encoding for integrase. Nested PCR sensitivity has been estimated at the level of 1 copy, while qPCR yielded 10 copies per 500 ng of DNA. PCR method in semi-nested format has been recently developed to detect EFV DNA in peripheral blood leukocytes of horses employing primers matching *pol* gene region [182].

## 6. Perspectives for Future Research

In sum, FVs, even if often compared to lentiviruses, show a unique performance among all retroviruses. So far there are many unknown aspects regarding their viral life cycle particularly with regards to the infected host, making it difficult to assess the risk of zoonotic or interspecies transmissions. But also on the host side not much is known about the transmission routes of FVs, the organ distribution and any interactions with other pathogens or even FVs’ role as a cofactor for known diseases. Also, there is a lot of leeway to make up regarding unsolved questions concerning the unusual Gag assembly, late reverse transcription with the consequence of DNA instead of RNA as genetic material in the virions as well as the interactions with host restriction factors. Besides counteraction of APOBEC3, the accessory protein Bet might play an even more important role in replication, infection and viral persistence than expected. In addition, little is known about FV and its host immune interaction and especially about the primary target cells. 

However, as stated before, FVs behave *in vivo* partly tremendously different as compared to *in vitro* observations. Therefore it is important to understand the different FVs within their corresponding hosts. 

Notwithstanding the fact that extensive work has been done on primate/human FV research it should be emphasized how important the outcome of animal FVs research could possibly be. Of course, it is to be expected that SFVs are transmitted to humans, and the ecological and social shift of the world’s population create a situation where viruses can easily reach distant areas and might cause epidemiological threat. In line with this, the justification for working with SFVs counts all the more for work on animal FV research. Overall, animal FVs appear to be more “prototypic” and therefore better suitable for basic comprehensive FV research as PFV itself. Moreover, animal FVs also exhibit the potential for interspecies as well as zoonotic transmissions. Though humans have closer contact with animals than simians (farming, domestic animals, zoo, butchering and consumption of various animal derived products) clear reports of transmissions are lacking. Explanations could be either the low genetic identity between humans and animals, compared to simians, a different route of transmission or low resolution power in the diagnostic assays used so far. Therefore improving our knowledge about the animal FVs will enable us to enhance current serological and molecular diagnostic methods as well as to provide molecular knowledge about the foamy viral life cycle. Subsequently these results might be transposable to enrich PFV and primate FV research. Furthermore, this effort will also yield new animal models and a deeper understanding for FV-host interactions which is desperately needed for establishing FV vectors for gene therapy. 
